# Production of a high-efficiency cellulase complex via β-glucosidase engineering in *Penicillium oxalicum*

**DOI:** 10.1186/s13068-016-0491-4

**Published:** 2016-03-31

**Authors:** Guangshan Yao, Ruimei Wu, Qinbiao Kan, Liwei Gao, Meng Liu, Piao Yang, Jian Du, Zhonghai Li, Yinbo Qu

**Affiliations:** State Key Laboratory of Microbial Technology, Shandong University, Jinan City, 250100 Shandong Province China; National Glycoengineering Research Center, Shandong University, Jinan City, 250100 Shandong Province China; Department of Bioengineering, Qilu University of Technology, Jinan City, 250353 Shandong Province China

**Keywords:** *Penicillium oxalicum*, Biofuel, β-glucosidase, Genetic engineering

## Abstract

**Background:**

*Trichoderma reesei* is a widely used model cellulolytic fungus, supplying a highly effective cellulase production system. Recently, the biofuel industry discovered filamentous fungi from the *Penicillium* genus as a promising alternative to *T*. *reesei*.

**Results:**

In our study, we present a systematic over-expression analysis of nine β-glucosidase encoding genes in the wild-type strain 114-2 of *Penicillium oxalicum.* We found that the over-expression of BGL1, BGL4, or BGL5 significantly enhanced both β-glucosidase activity and hydrolysis efficiency of the enzyme system on filter paper. We utilised two strategies to over-express β-glucosidase in the strain RE-10 that—although over-producing cellulase, does so at the cost of the cellulase mixture deficiency. The constitutive promoter of gene *pde_02864* encoding 40S ribosomal protein S8 was used to over-express three β-glucosidases: BGL1, BGL4, and BGL5. We found that all mutants show significantly enhanced levels of β-glucosidase at transcriptional, protein, and activity levels. Furthermore, the inducible promoter from *bgl2* was used to conditionally over-express the β-glucosidases BGL1 and BGL4. Surprisingly, this induced expression strategy enables significantly improved expression efficiency. The BGL1 over-expressing mutant I1-13 particularly improved the β-glucosidase activity at a factor of 65-folds, resulting in levels of up to 150 U/ml. All our BGL over-expression mutants displayed significant enhancement of cellulolytic ability on both microcrystalline cellulose and filter paper. In addition, they substantially reduced the enzyme loads in the saccharification of a natural lignocellulose material delignified corncob residue (DCCR). The mutant I4-32 with over-expression of BGL4 achieved the highest glucose yield in the saccharification of DCCR at only 25 % enzyme load compared to the parental strain RE-10.

**Conclusions:**

In summary, genetically engineering *P*. *oxalicum* to significantly improve β-glucosidase activity is a potent strategy to substantially boost the hydrolytic efficiency of the cellulase cocktail, which will ultimately lead to a considerable reduction of cost for biomass-based biofuel.

**Electronic supplementary material:**

The online version of this article (doi:10.1186/s13068-016-0491-4) contains supplementary material, which is available to authorized users.

## Background

The conversion of plant-based biomass into biofuel and chemicals is a promising course to absorb the upcoming shortage of fossil fuels without any serious environmental problems. Cellulose is the major building block of plant biomass [1]. Fungal cellulases transform plant polysaccharides into fermentable sugars, which in turn determine the cost of plant-based biofuel. Throughout the enzyme industry, the champion for the production of cellulase cocktail is *Trichoderma reesei* [2–4]. However, an increasing number of publications demonstrate better hydrolysing lignocellulose performance of the cellulase enzyme mixture obtained from *Penicillium* strains. Thus, *Penicillium* strains are considered a potent alternative to the *T*. *reesei* cellulase cocktail [5].

Three *Penicillium oxalicum* strains in particular, secrete a more balanced lignocellulose-degrading enzyme system and display higher β-glucosidase activity compared to *T*. *reesei.* These are: *P*. *oxalicum* 114-2 [6], *P*. *oxalicum* GZ-2 [7], and *P*. *oxalicum* 16 [8]. *P*. *oxalicum* 114-2 has been studied in our laboratory for more than 30 years. Multiple cycles of mutagenesis and screening led to the carbon catabolite repression-resistant mutant JU-A10-T with a volume productivity of 160 IU/Lh. For the last 20 years, this mutant produces cellulase enzyme preparations at industrial scale in China [9]. Recently, its genome has been sequenced, providing valuable information to mine novel components that play key roles in plant cell wall deconstruction [6]. Furthermore, the signal transduction, inducer transportation, and transcription regulation mechanism of the cellulase gene expression of *P*. *oxalicum* has been partially uncovered. The sequenced genome, high-efficient gene knock-out techniques, and system biology (RNA-seq and proteomics) are the methods that have led to this advance. Gene disruption analysis allowed the identification of the three major cellodextrin transporters CdtC, CdtD, and CdtG that play crucial roles in cellulase induction [10]. Hu et al. [11] revealed that the G protein-cAMP signaling pathway downregulates the expression of cellulolytic genes. Furthermore, a single-gene deletion library has been established for 470 transcription factors and 15 novel and 4 major transcriptional regulators. Their roles in the cellulase expression regulatory network have been identified and characterized [12]. The Reconstruction of Expression Regulatory Network (REXRN) technology has been developed as a new strategy to engineer fungi that enhance cellulase and protein production. One mutant in particular (RE-10 from REXRN) displayed a drastic increase in cellulase and hemicellulase production, and produced even higher values compared to the industrial strain JU-A10-T [13]. However, the β-glucosidase of RE-10 has not yet been improved to the same level as other inducible cellulases. This makes further improvement of its β-glucosidase activity necessary.

Beta-glucosidase is the rate-limiting enzyme because it is responsible for the final step of lignocellulose hydrolysis and converts cellobiose as well as short cellodextrins into glucose [14]. Fungal β-glucosidases are classified into glycosyl hydrolases families 3 and 1 (GH3 and GH1) [15, 16]. Different proteins exhibit various characteristics in specific activity, substrate specificity, stability and others in the same family of β-glucosidase [17]. The genome of *P*. *oxalicum* includes 11 β-glucosidase encoding genes, of which only β-glucosidase 1 (BGL1) and β-glucosidase 2 (BGL2) have been identified [18, 19].

To solve the shortage of β-glucosidase in *T*. *reesei* enzyme cocktails, a time-consuming and laborious process is used to supplement heterologous β-glucosidases from *Aspergillus* into commercial cellulase preparations [14]. We systemically screened the β-glucosidases via over-expression analysis and found three β-glucosidases from *P*. *oxalicum* to have a promising enzymatic performance. The three BGLs were over-expressed either constitutively, or inductively by means of two different types of promoters. We obtained a number of high-yield β-glucosidase producers and their β-glucosidase yields were elevated from twofold to 65-fold. Microcrystalline cellulose, filter paper and corncob residue were used as substrates to assess hydrolysis efficiency of the enzyme complex.

## Results and discussion

### Systematic over-expression analysis of nine β-glucosidases in *P*. *oxalicum* 114-2

The β-glucosidases of *P*. *oxalicum* are encoded in a total of 11 genes. Among these, four β-glucosidases belong to the glucoside hydrolase family 1 (GH1) with the other seven BGLs belonging to GH3. Using multiple algorithms, we found the signal peptides for five β-glucosidases (Table 1), implying a high likelihood for these enzymes to secret extracellularly. However, of these, only BGL1 had been detected in a previous secretome analysis [6]. In order to systematically examine all β-glucosidases in *P*. *oxalicum*, we performed a systematic over-expression analysis, using a constitutive promoter from gene *pde_02864* to drive BGL expression. We obtained 9 out of 11 BGLs over-expression transformants, while over-expression mutants for the remaining two genes were not available after several rounds of genetic transformation. The promoter of gene *pde_02864* that decodes the 40S ribosomal protein S8, has previously been identified as a strong and constitutively promoter [12]. We then evaluated the *p*NPGase activities and filter paper hydrolysis rate of all the obtained β-glucosidase over-expression mutants (obtaining at least two transformants for each gene). Both the BGL(X) over-expression mutants and RE-10 were cultured in cellulose medium for enzyme production. Our results (Table 1) show, that the over-expression of seven BGLs significantly increased *p*NPG activity. Among these, over-expression of BGL1, BGL4, BGL5, and BGL7 resulted in more than twice β-glucosidase activities of that of WT. In parallel, the crude enzyme from BGL1, BGL4, and BGL5 over-expression mutants significantly improved the hydrolysis rate of filter paper (see Table 1). However, over-expression of both *bgl3* and *bgl8* slightly reduced the extracellular β-glucosidase (less than twofold) and FPase activities. BGL8 is an intracellular β-glucosidase and thus hydrolyze intracellular cellobiose into glucose, which enhancing the repression and alleviating the induction of cellulolytic system. BGL3 had been expressed and purified in *Pichia pastoris*. However, the BGL3 did not showed activities against both *p*NPG and salicin in vitro (data not shown), and their roles (BGL3 and BGL8) in regulation of the expression of cellulase and β-glucosidase required further investigation. Therefore, BGL1, BGL4, and BGL5 are considered as the most efficient β-glucosidases and hence, we targeted them in the following strain engineering for a cellulase high-producer.

**Table 1:** Systematic screening the major β-glucosidases

BG(X)	Gene ID	Signal peptide	GH family	Fold change (transcript level)	Fold change (*p*NPGase activity)	Fold change (filter paper hydrolysis rate)
BGL1	PDE_02736	Yes	GH3	3.93	2.023	1.37
BGL2	PDE_00579	No	GH1	4.48	1.537	0.68
BGL3	PDE_01277	Yes	GH1	40.4	0.650	0.92
BGL4	PDE_01565	Yes	GH3	492.1	9.335	1.20
BGL5	PDE_02905	Yes	GH3	132.0	6.439	1.10
BGL6	PDE_09019	Yes	GH3	17.8	1.797	0.89
BGL7	PDE_02108	No	GH3	79.6	2.427	0.73
BGL8	PDE_04859	No	GH1	68.0	0.868	0.89
BGL9	PDE_03485	No	GH3	13.2	0.868	0.90

*Yes* with signal peptides, *No* without signal peptides

Similar to previous cellodextrin transporter studies [10], gene knockout analysis is unhelpful for identification of the essential genes that are responsible for β-glucosidase activity, due to high functional redundancy [20]. We propose that the single-gene over-expression analysis we establish in this study is effective to discriminate the important β-glucosidases, and other high-redundant genes (or gene families) in filamentous fungi.

### Sequence and phylogenetic analysis of three major β-glucosidases from *P*. *oxalicum* and other cellulolytic fungi

**Fig. 1:**
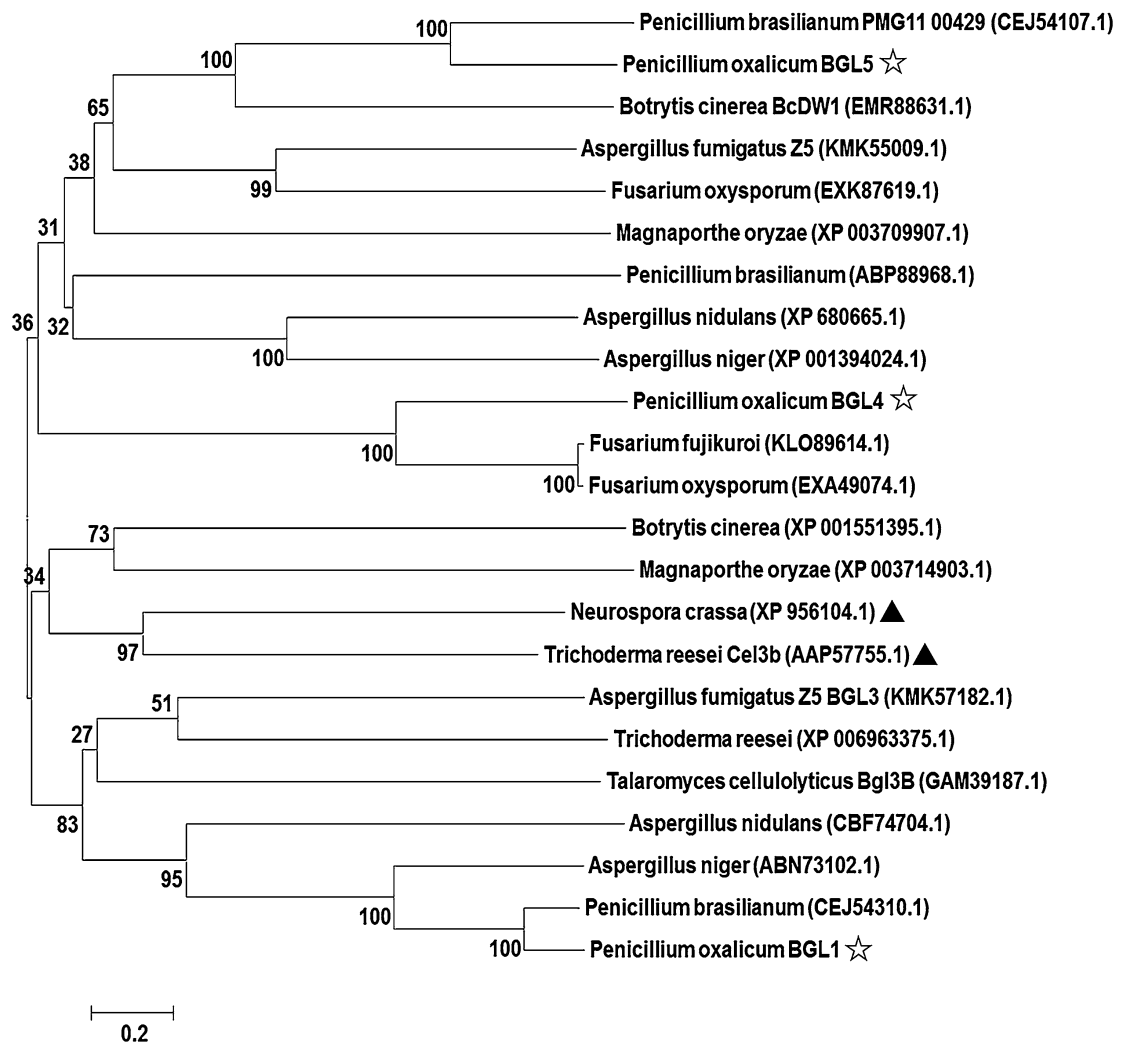
Phylogenetic analysis of BGL1, BGL4, and BGL5 proteins in *P*. *oxalicum* and their orthologs from other filamentous fungi. Construction of phylogenetic tree was based on neighbor-joining analysis and full-length amino acids for all proteins were used to generate the tree with the software MEGA 6.0. *Filled triangle* indicates the characterized BGL(X) homolog in *N*. *crassa* or *T*. *reesei*. *Star* indicates that the BGL(X) from *P*. *oxalicum*

BGL1 was the most conserved extracellular β-glucosidase, and its homolog could be found in many cellulolytic fungi, such as *P*. *brasilianum*, *T*. *reesei*, *Neurospora crassa*, *Aspergillus nidulans*, *A*. *fumigatus*, *A*. *niger* and *Talaromyces cellulolyticus*, and plant pathogens with a capacity of degrading plant cell wall, including *Botrytis cinerea*, *Magnaporthe oryzae* (Fig. 1), implying its essential role in lignocellulose deconstruction in the natural system. Orthologs of BGL5 can be found in most of the above cellulolytic fungi, with the exception of *T*. *reesei* and *N*. *crassa* (Fig. 1). Interestingly, BGL4 could only be identified in *P*. *oxalicum* and the *Fusarium* genus and forms a clade separate from BGL1 and BGL5 (Fig. 1). This suggests an evolutionary development from gene expansion. However, most of the β-glucosidases from the *Fusarium* genus remain unidentified.

All three efficient β-glucosidases are classified as members of the GH3 family (Table 1), and have three functional domains: the N-terminal domain, the C-terminal domain, and a fibronectin-like domain with unknown function (see Additional file 1: Figure S1). According to a previous study, the former two domains are likely forming a catalytic pocket [21].

### Over-expression of β-glucosidases in RE-10 using a constitutive promoter

The cellulase over-producer RE-10 has previously been obtained by redesigning the regulatory pathway of cellulase gene expression in *P*. *oxalicum*. As a result, most enzyme proteins and their corresponding activities, were significantly enhanced, with the exception of BGL1 and *p*NPGase activity remaining unaffected [13]. Therefore, improvement of the β-glucosidase activity in RE-10 was the main goal of our study. Via systematic screening, we identified BGL1, BGL4, and BGL5 as the most promising β-glucosidases for industrial scale *Penicillium* cellulase production.

**Fig. 2:**
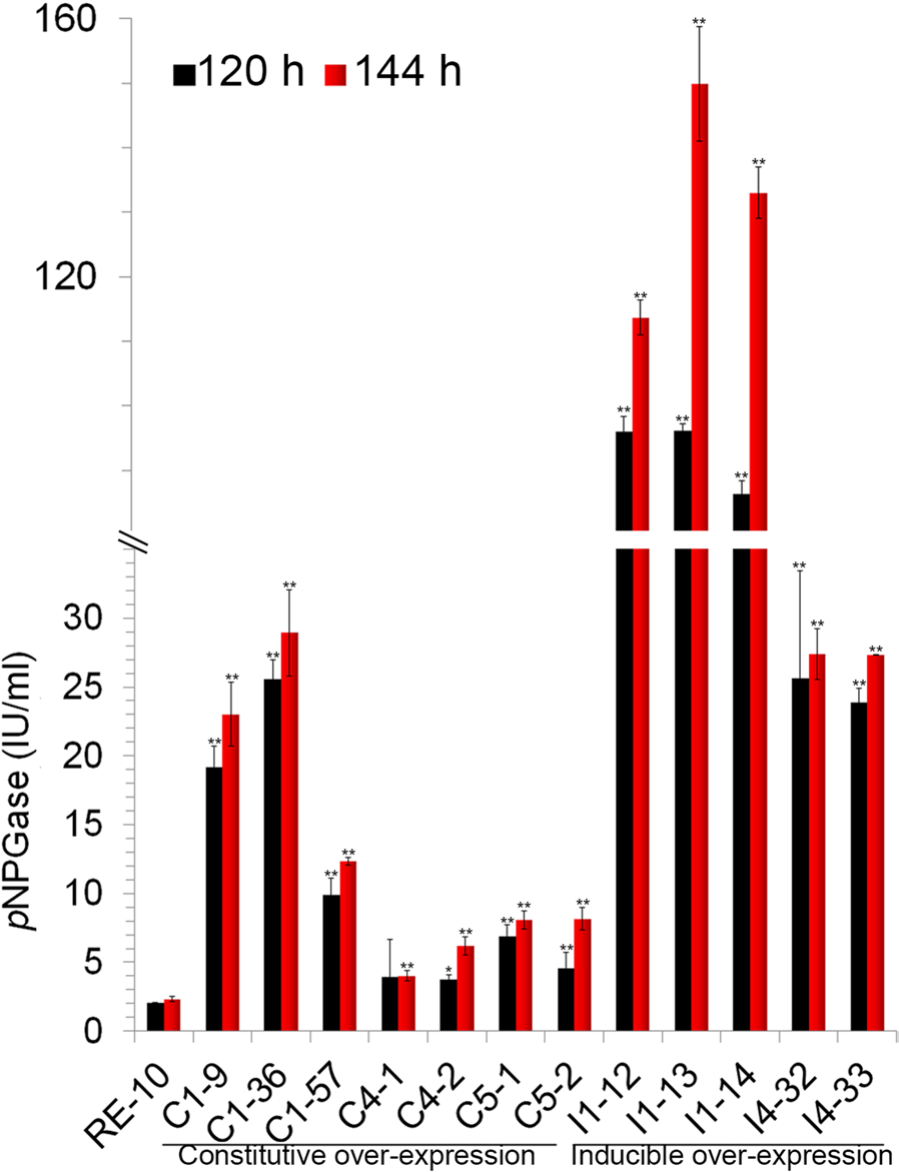
Comparative analysis of *p*NPGase activity between BGL(X) over-expression mutants and the parental strain RE-10. The culture supernatants of 12 BGL(X) over-expression mutants and the parental strain RE-10 were sampled at 120 and 144 h. β-Glucosidase activity was measured with the *p*NPG as the substrate. Data are the means of three biological replicates and *error bars* show the standard deviation. *Asterisks* indicates the significantly difference between the mutant and the RE-10 (*p ≤ 0.05; **p ≤ 0.01)

The three β-glucosidases encoding genes *pde_02736*, *pde_01565* and *pde_02905*, were driven by the promoter of *pde_02864* and expressed in the background of RE-10. Following single spore isolation, three BGL1, two BGL4, and two BGL5 over-expression mutants were selected for further analysis. All the putative over-expression mutants showed increased *p*NPGase activity. As shown in Fig. 2, all the BGL over-expression mutants also had a significant increase (*p* ≤ 0.01) in β-glucosidase activity. Three BGL1 over-expression mutants increased *p*NPGase activity over 10, 12, fivefold, respectively, compared to the parental strain RE-10. Remarkably, the *p*NPGase of one of them (C1-57) reached up to 30 IU/ml, which is almost the highest value reported throughout all *Penicillium* strains with the ability for lignocellulose degradation. This possibly correlates with the fact that BGL1 has a lower Km than the BGLs from other fungi [18]. In addition, both BGL4 and BGL5 over-expression mutants displayed twofold higher β-glucosidase activity over RE-10 at 120 and 144 h. Cellobiose is proposed as the natural substrate of β-glucosidase and is the product of CBH and EG during cellulose hydrolysis. To further illuminate their enzymatic performance of the BGL(X) over-expression mutants, we examined their cellobiose hydrolysis activity, and found that all the BGL(X) over-expression mutants improved glucose release from cellobiose by more than tenfold, which is consistent with the above *p*NPGase activity analysis (see Additional file 2: Figure S2).

### Inducible over-expression β-glucosidases in RE-10

Intracellular β-glucosidase BGL2 in *P*. *oxalicum* has previously been identified as a negative regulator for cellulase gene expression [19]. Interestingly, its expression pattern is similar to other inducible cellulase genes, such *cel7A*-*2*, *cel5B* and *cel7B*. As a result, the *bgl2* promoter was designed to over-express secretory β-glucosidases. Multiple rounds of transformation and screening led to identification of three mutants for BGL1 over-expression (I1-2, I1-3, and I1-4), and two mutants for BGL4 over-expression (I4-32 and I4-33). Despite considerable effort, no *sur*-resistant transformants for BGL5 could be obtained. Notably, all inducible mutants (I1-12, I1-13, I1-14, I4-32, and I4-33) displayed drastic increases in β-glucosidase activity towards both *p*NPG and cellobiose, compared to their respective constitutive over-expression mutants. One of them in particular (I1-13) has a *p*NPGase activity of 150 U/ml (Fig. 2) and a cellobiose activity of 36 U/ml (see Additional file 2: Figure S2). These activities are more than 65- and 93-fold higher than those of RE-10. In *T*. *reesei*, the strong *cbh1* promoter was previously used to drive *bgl1* expression, but the recombinant strain produced a *p*NPGase activity of only about 30 U/ml [22], which is significantly lower compared to that found in our study.

Genetic engineering of the regulatory machinery of cellulolytic genes is a promising strategy to create protein over-producers. Cellobiohydrolase, endo-glucanase, and other proteins that play significant roles in cellulose degradation, were all synchronously improved in our previous work, except for β-glucosidase [13]. Another pathway-specific regulatory mechanism for the expression of β-glucosidase is present in cellulolytic fungi and the β-glucosidase specific regulator BglR, which was found in *T*. *reesei* further strengthened this proposal [23]. As a consequence, additional improvement of β-glucosidase levels in the engineered cellulase over-producers is crucial for producing efficient as well as more balanced enzyme mixtures.

A recent study reports a strong cellulose induction of the gene *bgl2* and corresponding repression by glucose-mediated carbon catabolite repression (CCR) [12]. The expression pattern of this gene is highly synchronous with cellulase gene induction. Furthermore, the cellulase gene transcription activator ClrB activates its level, while the repressor CreA represses its level. Interestingly, both the *bgl2* [20, 24] and its regulators CreA/Cre1 [25, 26] are highly conserved in the cellulolytic fungi *N*. *crassa* and *T*. *reesei*. Our data further highlights the *bgl2* promoter to be a highly efficient and inducible promoter in *P*. *oxalicum* and likely also in other cellulolytic fungi, such as *N*. *crassa* and *T*. *reesei*. It will be a particularly valuable tool for strain genetic engineering, to conditionally super-express other essential enzymes or proteins.

The sugars produced during the fermentation process were examined by high-performance liquid chromatography (HPLC). As expected, cellobiose was only detected in RE-10, but not in the other β-glucosidase over-expression mutants (see Additional file 3: Figure S3). In fact, the cellobiohydrolase and endo-glucanase activities were slightly enhanced in the β-glucosidase mutants (data not shown). The expression level of CBH and EG encoding genes were further analyzed (Additional file 4: Figure S4). The result showed that the expression levels of major CBH encoding gene *cbh1* in C1-9, and major EG gene *eg1* in C1-9, I1-12 and C4-1 were up-regulated significantly. On the other hand, previous assumptions pointed towards cellobiose to be the inhibitor of both cellobiohydrolase and endo-glucanase [27]. As a result, it was suggested that over-expression of β-glucosidase might indirectly increase both cellobiohydrolase and endo-glucanase activities by removing the product feedback inhibition caused by cellobiose.

### Improvement of microcrystalline cellulose degradation via over-expression of β-glucosidases in RE-10

**Fig. 3:**
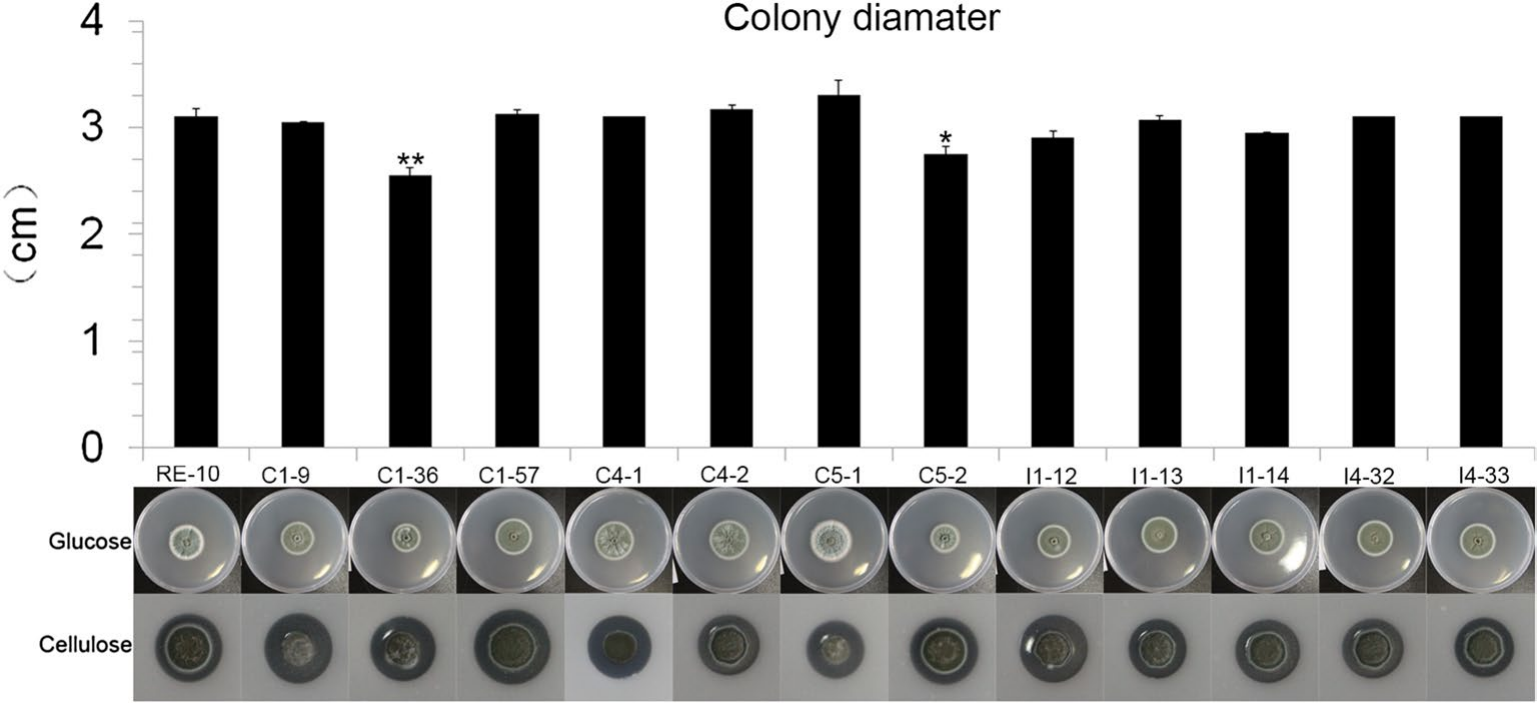
The effects of β-glucosidase over-expression on fungal growth and cellulolytic ability. Spores of 12 BGL(X) over-expression mutants and RE-10 were inoculated on the glucose and cellulose plates and cultured for 6 days. The diameters of fungal colonies on glucose were measured and displayed in *bar column* (*up*) and cellulolytic zones represented their cellulolytic abilities (*down*)

We assayed fungal hyphae extension rates of all mutants and used higher concentration cellulose in the plate medium to evaluate cellulolytic ability. Hyphae extension speeds and colony diameters of most of the BGL mutants resemble that of RE-10 (Fig. 3), which indicates that over-expression of BGLs did not affect fungal growth. However, the mutants C1-36 and C5-2 decreased fungal growth rate. A possible reason for this could lie in the genomic sites of expression cassette insertion, so we removed these two strains from further analysis. The cellulolytic zones of 8 over-expression strains increased significantly compared to RE-10, including C1-9, C1-36, C1-57, C5-1, I1-12, C4-1, I1-14, I4-32 and I4-33 (Fig. 3). This correlates with our results for the β-glucosidase activity assay. Our data demonstrates that over-expression of β-glucosidases significantly improves the fungal cellulolytic ability.

### Up-regulation of BGL(X) in the mutants at both transcription and protein levels

**Fig. 4:**
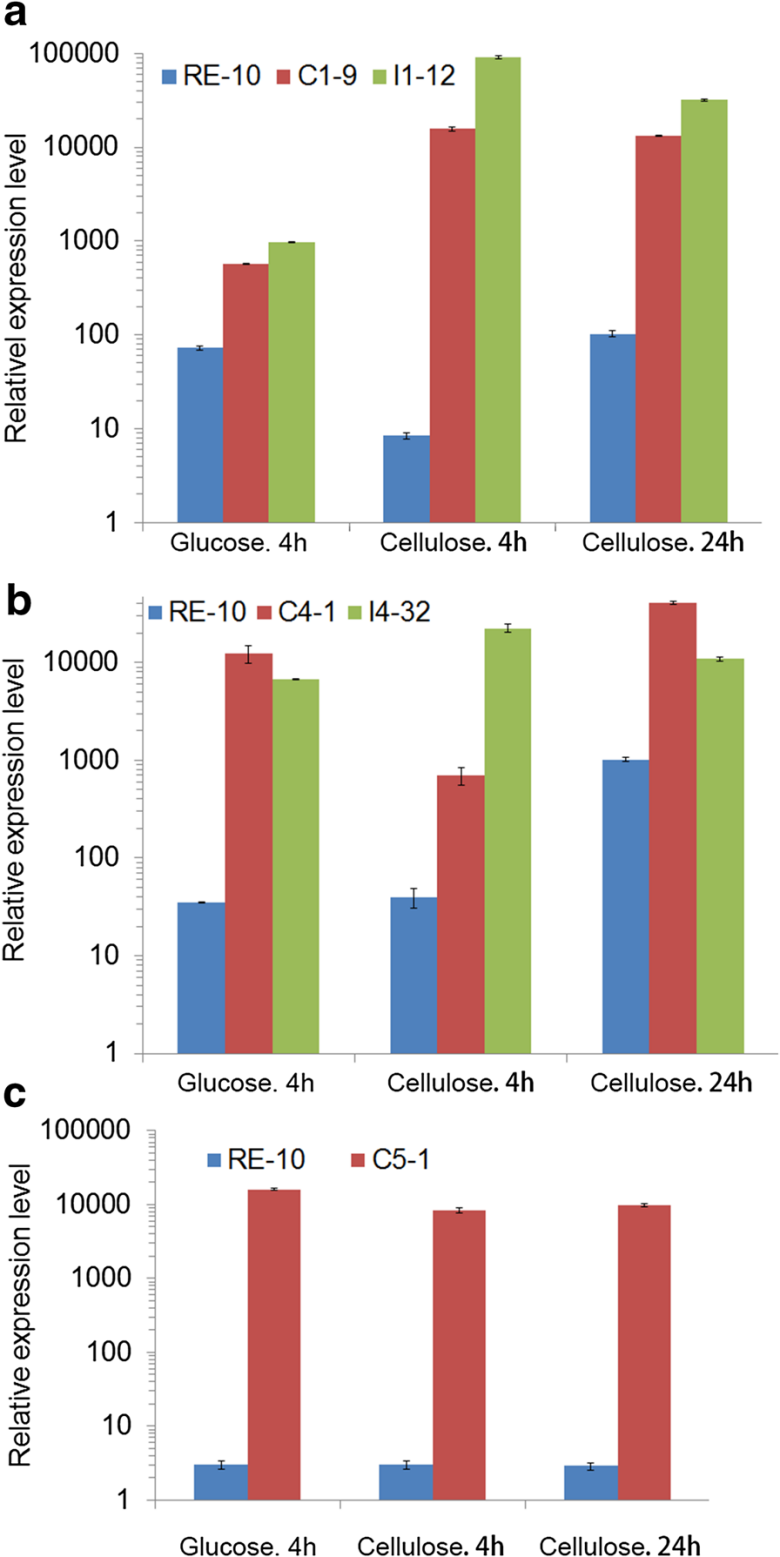
qPCR analysis the transcription levels of the BGL(X) gene in the corresponding mutants. The transcription level of *bgl1* (**a**), *bgl4* (**b**), and *bgl5* (**c**)were determined at the 4 or 22 h for cellulose induction or at 4 h for the culture on glucose. The means of three biological replicates was showed and the *error bars* indicated the standard deviation

**Fig. 5:**
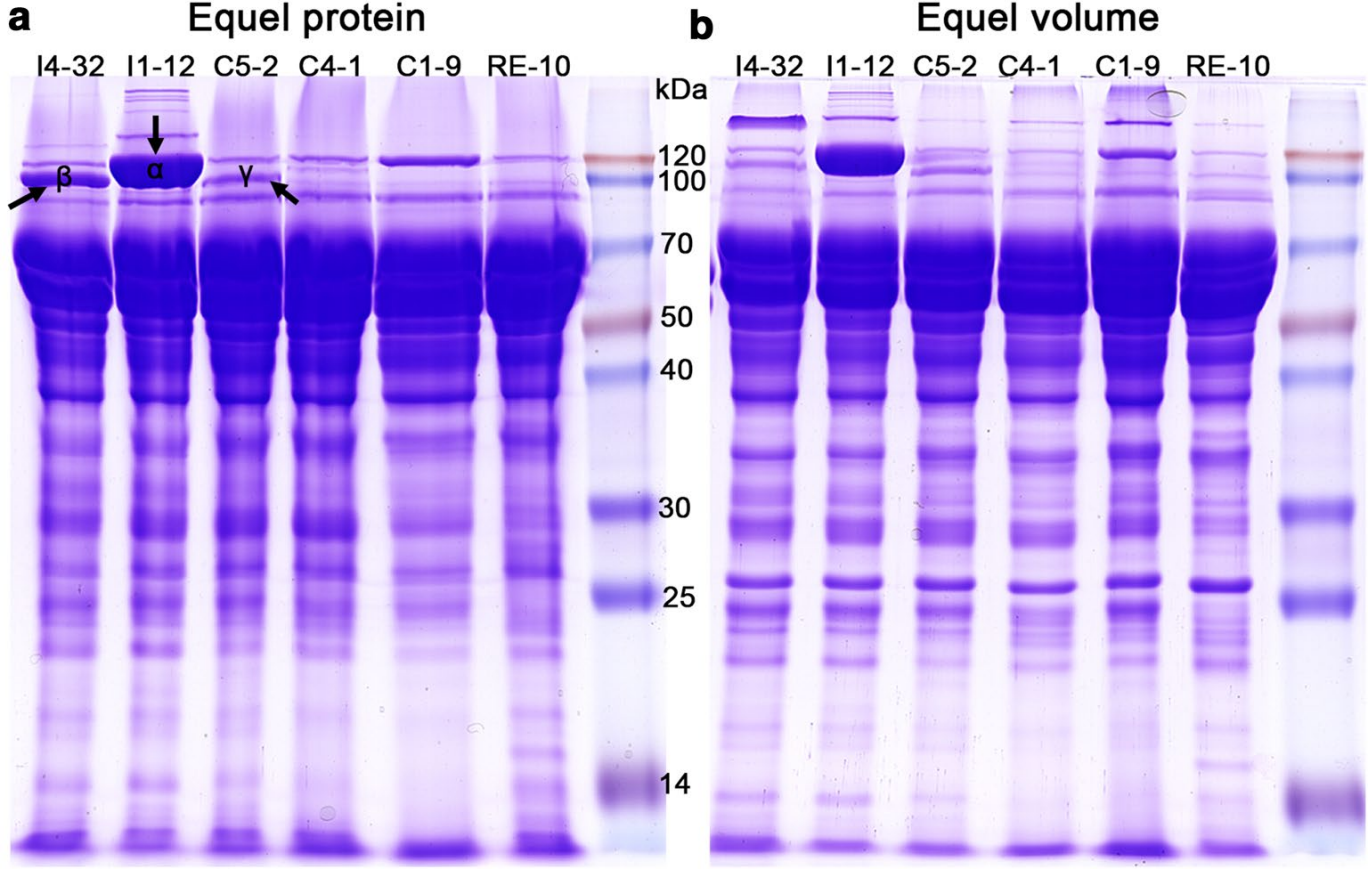
The increase of β-glucosidase at the protein level analyzed by SDS-PAGE and mass spectrometry. Equal amount (**a**) and equal volume (**b**) of crude enzymes of five BGL(X) over-expression mutants and RE-10 were used for SDS-PAGE analysis and MS identification (*α* BGL1; *β* BGL4; *γ* BGL5)

Although all the above mutants have been verified by PCR using specific primers (data not show), the transcript abundance of β-glucosidase genes has not been examined. Fluorescence quantitative polymerase chain reaction (qPCR) was performed to investigate the transcript abundance of BGL(X). The results are shown in Fig. 4. Both the C1-9 and I1-12 strains substantially up-regulated their *bgl1* expression levels in both glucose and cellulose conditions. Markedly, after cellulose induction for 4 h, the transcriptional level of *bgl1* in C1-9 increases about 1000-fold, and in I1-12 up-regulates 10,000-fold. The expression level of *bgl5* in C5-1 was also greatly up-regulated with values of up to 5000-fold in comparison to RE-10. The expression level of *bgl4* in C4-1 and I4-32 was also significantly elevated. Our data confirmed that all of the β-glucosidase mutants we used, improved the corresponding β-glucosidase at the transcription level. Given that the expression levels of all three *bgl* genes were comparable in their over-expression strains, we assumed that higher specific activity and substrate affinity of BGL1 contributes to higher β-glucosidase activity in the *bgl1*-over-expressing strains C1-9, C1-36, C1-57, I1-13, and I1-14. Furthermore, the SDS-PAGE and MS analysis confirmed that the BGL bands were significantly improved compared to those of RE-10 in the over-expression mutants (Fig. 5). The BGL1 amount of Il-12 in particular, is more abundant compared to that of CBHI (PDE_07945), which is the dominant protein in the cellulolytic secretome [6, 28, 29].

The regulation model of the three β-glucosidases was analyzed based on three kinds of different promoter-driven expression patterns. Three different promoters were from gene *bgl*(x), *pde_02864* and *bgl2*, respectively. The expression pattern of BGL1 and BGL4 was similar: low-level expression under glucose and high-level expression when induced by cellulose (Fig. 4 and Additional file 5: Figure S5), whose regulation model resemble CBH and EG. However, the *bgl5* displayed constitutive expression and independent of carbon source (Fig. 4c).

### Over-expressing β-glucosidases enhanced the filter paper hydrolysis

**Fig. 6:**
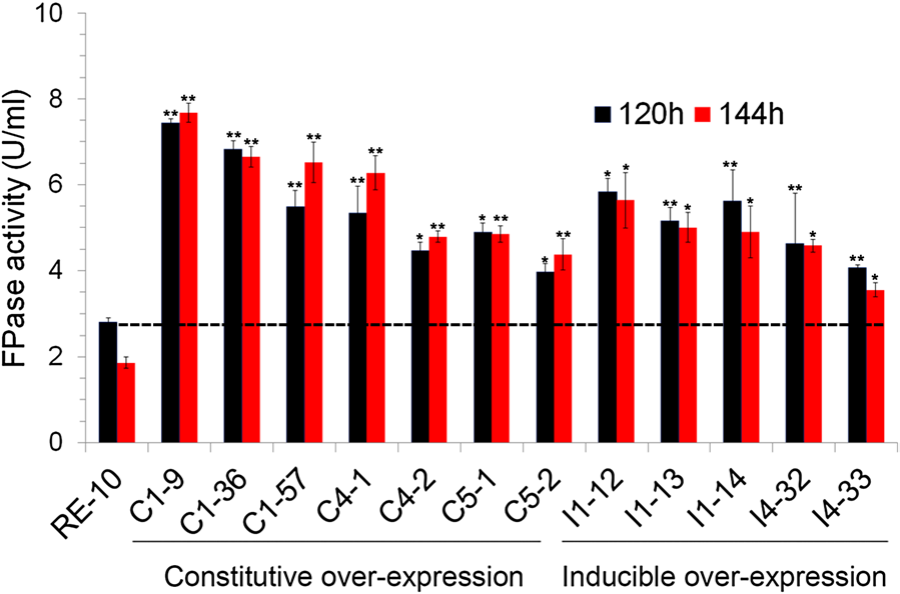
Analysis of hydrolysis ability of crude enzymes against filter paper. The culture supernatants of the BGL(X) over-expression mutants and the parental strain RE-10 were sampled at 120 and 144 h. The released glucose from filter paper was measured. Data are the means of three biological replicates and *error bars* show the standard deviation. *Asterisks* represents the significantly difference between the mutant and the RE-10 (*p ≤ 0.05; **p ≤ 0.01)

For the process of lignocellulose hydrolysis, β-glucosidases affect the last step and are also the important rate-limiting enzymes. To further test the cellulolytic ability of the above β-glucosidase over-expression strains, filter paper was used as substrate. The result is shown in Fig. 6. All β-glucosidase mutants exhibit a significant increase in glucose release compared to RE-10. This suggests that high β-glucosidase activity has a strong impact on filter paper hydrolysis (Fig. 3). Most of β-glucosidase over-expression mutants increase the FPase activity over twofold compared to RE-10. This result further confirmed the significant role of β-glucosidase as rate-limiting enzyme in the lignocellulose-glucose conversion. The constitutive β-glucosidase over-expression strain had a slight higher activity compared to those of corresponding inducible mutants. Especially, C1-9 displayed the highest FPase activity among all with up to 8.0 U/ml (Fig. 6).

### Cellulase enzyme complex with β-glucosidase over-expression showed higher hydrolysis efficiency against delignified corncob residue (DCCR)

**Fig. 7:**
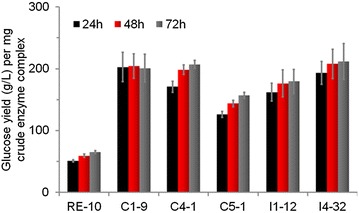
Saccharification of DCCR with the crude enzymes of BGL(X) over-expression mutants and the parental strain RE-10. The crude enzyme complex from *P*. *oxalicum* RE-10 and BGL(X) over-expression mutants were used for saccharification of DCCR. Culture supernatants of 10 FPA were mixed with DCCR. Released glucose concentration were measured every 24 h. Data are the means of three biological replicates and *error bars* show the standard deviation

In nature, neither cellulose, nor filter paper is the staple lignocellulose materials. In order to clarify the hydrolysis ability of the cellulase mixture of all BGL(X) mutants on natural cellulosic materials, the enzyme mixture from their supernatants were used to hydrolyze DCCR. The released glucose for DCCR gradually increased with time from 24 to 72 h. With equal FPU loading, all strains produced similar amounts of glucose from hydrolyzing DCCR, with approximately 40 g/l during 72 h. However, we only required 27 % of enzyme loadings for C4-1, 36 % for C5-1, 31 % for I1-12, and 38 % for I4-32 to obtain the same hydrolysis rates compared to RE-10. These results signify that these β-glucosidase over-expression mutants noticeably reduce total crude enzyme loading, and therefore reduce enzyme cost. The amount of released glucose per mg protein is illustrated in Fig. 7. The data clearly shows that all the BGL(X) mutants produce more than twice or three times the glucose (from 158 to 212 g glucose/mg crude enzyme complex) at the equivalent protein basis compared to the parental strain RE-10 (65 g glucose/mg crude enzyme complex) (Fig. 7). Although the released glucose of C1-9 was slightly less than that of RE-10 at 72 h, only 22 % protein load was supplemented compared to RE-10. We assume that the mild decrease of hydrolysis rate in C1-9 to be due to a lack of other proteins that play a role in the DCCR hydrolysis. Noteworthy, the strain I4-32 displayed significantly higher glucose yield compared to that of RE-10 at lower protein loading. Engineering the β-glucosidase as described in this study contributes to manufacture high-efficiency cellulase complex.

A growing number of publications report the reduction of enzyme loadings in the saccharification process and supplementing β-glucosidase undoubtedly becomes one of the most efficient strategies [22, 30, 31]. In this study, the β-glucosidases were directly improved by strain engineering, which is both time and labor-saving. Lytic polysaccharide monooxygenase (LPMO) [32, 33], expansion and expansion-like proteins [34], and other proteins [35] were reported to play important and synergetic roles in lignocellulose degradation. Their supplementation could significantly improve the lignocellulose-to-glucose conversion rate. With the highly efficient promoters found in this study, we were able to enhance their levels by over-expressing those proteins, and thereby ultimately reduce the enzyme cost.

## Conclusions

This study presents over-expression analysis of nine β-glucosidase genes in wild-type *P*. *oxalicum* and identifies three major players of lignocellulose degradation: BGL1, BGL4 and BGL5. Two different types of promoters were used to over-express the three β-glucosidases in the cellulase over-producer RE-10. Compared to the parental strain, we observed significant boost in beta-glucosidase activity for all recombinant strains obtained. The inducible promoter from gene *bgl2* is particularly more efficient in activating the *bgl1* or *bgl4* gene expression and is proposed to be a potentially valuable tool to conditionally over-express other essential genes in *P*. *oxalicum* as well as other cellulolytic fungi. The crude cellulase mixture from these β-glucosidase over-producers was significantly more efficient in hydrolyzing microcrystalline cellulose, filter paper, and DCCR compared to that of RE-10. This substantially reduced the enzyme cost of lignocellulose-based biofuel.

## Methods

### Strains and culture conditions

*P*. *oxalicum* 114-2 (CGMCC 5302), the cellulase high-producer RE-10 [13] and all mutants we obtained from these two, were maintained on malt extract agar. The glucose and cellulose medium comprised 1× Vogel’s salts [51] and 2 % glucose or microcrystalline cellulose as the sole source of carbon. The enzyme-producing medium was composed as follows: corn cob residue (2.0000 %), cellulose (0.6000 %), wheat bran solid (4.6571 %), soybean cake powder plate (1.0000 %), (NH_4_)_2_SO_4_ (0.2000 %), NaNO_3_ (0.2789 %), urea (0.1000 %), KH_2_PO_4_ (0.3000 %), and MgSO_4_ (0.0500 %). To induce enzyme production, 10^7^ spores were pre-cultured for 22 h in glucose medium and subsequently transferred into 1× Vogle’s solution devoid of any carbon source, where it stayed for 2 h. As a final step, the cultures were then transferred to cellulose or enzyme-producing media at 30 °C, and 200 rpm for 144 h. All samples were collected at the respective time points as described in the text.

### Bioinformatics analysis

The sequences of BGL1, BGL4, and BGL5 were used as the search queries in the NCBI database (http://www.ncbi.nlm.nih.gov/). All BGL sequences form *P*. *oxalicum* and their orthologs from other fungi were downloaded from NCBI’s protein database. The software package MEGA 6.0 [36] was used to construct phylogenetic trees with neighbor-joining algorithms and using 500 bootstrap replications. The domain predictions for BGL1, BGL4, and BGL5 were performed using Pfam 28.0 (http://pfam.xfam.org/) and the signal peptide prediction was based on SignalP 3.0 Server (http://www.cbs.dtu.dk/services/SignalP-3.0/).

### Generation of the BGL(X)-over-expression mutant

Throughout this study, we used high-fidelity FastPfu DNA polymerase (TransGen Biotech, Beijing, China) for all PCR. PEG-mediated fungal transformation protocols were performed following published protocol [37]. The constitutive promoter from gene *pde_02864* was PCR-amplified with primers DF and DR, using the *P*. *oxalicum* 114-2 genome as the template. We amplified the gene encoding regions of nine β-glucosidases from *P*. *oxalicum* 114-2 with 20 bp overlapping fragments including the promoter and the downstream *hph* cassette. The resistance cassette of *hph* was amplified with primers HPHsF and HPHsR, and via the plasmid *p*Silent-1 [38] template. These gene over-expression cassettes were fused in the order *pde_02864*_(p)_-*bglx*_(coding region)_-*hph* by Double-joint PCR [39] with nest primers DF2 and HPHsR. All the BGL(X) over-expression cassettes were gel-purified and transformed into the protoplast of *P*. *oxalicum* 114-2.

In order to generate the *bgl*(x) over-expression mutant in RE-10, the mark gene *hph* of the over-expression cassettes of BGL1, BGL4, BGL5 were replaced with a novel resistance gene *sur* [12] using the primer pair SurF + SurR. The inducible expression cassettes for BGL1 and BGL4 were generated by individually amplifying their encoding region with corresponding primers (see Additional file 6: Table S1) and fused with both the *bgl2* promoter and the mark gene *sur*. All the over-expression cassettes were concentrated by gel purification and transformed into *P*. *oxalicum* RE-10 protoplasts.

### Beta-glucosidase activity, SDS-PAGE, and HPLC assays

The fermentation broth was collected via centrifugation, and the aliquots of the supernatant were diluted to measure enzyme activity. To examine β-glucosidase activity, *p*NPG and cellobiose (Sigma, USA) were used as substrates. We conducted the enzyme reaction in acetate buffer (pH 4.8) at 50 °C for a total of 30 min, after which we added 10 % Na_2_CO_3_ to stop the reaction. *p*NP release was measured and the absorbance was read at 405 nm. The glucose level was measured with the Biosensor. One enzyme activity unit represents the amount of enzyme required to either produce one µmol glucose, or *p*NP per minute under the above condition. To measure the FPase activity, the enzyme reaction was conducted in 0.2 mol/L acetate buffer (pH 4.8) at 50 °C for a total of 60 min with 0.05 g Whatman No. 1 paper as the substrate. DNS method was used to quantify the released reducing sugars. A protein concentration assay was performed using a Bradford kit (Sangon Biotech, Shanghai, China). Three biological triplicates were performed throughout all described experiments. Both equal quality and volume of culture supernatants were performed for SDS-PAGE analysis, and the predicted β-glucosidase bands were excised for MALDI-TOF–MS identification. We measured the released sugars within the broth supernatants via LC-10 AD HPLC (Shimadzu, Japan) by a BioRad Aminex HPX-42A carbohydrate column (Bio-Rad, USA).

### Fungal growth, microcrystalline cellulose hydrolysis and qRT-PCR analysis

Equal volumes of conidia (10^4^ per ml) of all *bgl*(x) mutants as well as the parental strains RE-10 were spotted on a solid plate using 2 % glucose or 3 % ball-milled cellulose at 30 °C for a total of 6 days, after which, they were photographed. The diameters of all colonies on glucose plates were measured, and this was used to evaluate fungal growth. Two biological triplicates were performed in the analysis. For RNA extraction, we inoculated the spores into glucose medium and pre-culturing them for 22 h, followed by a starvation period of 2 h and transferral into cellulose medium at 30 °C and 200 rpm for a total of 4 h. The RNAiso™ reagent and PrimeScript RT Reagent Kit (TaKaRa, Japan) were used to extract RNA and to synthesize cDNA, respectively, following the manufacture’s description. We performed the qPCR on LightCycler equipment as previously described [10]. We used the expression level of actin as the internal control for data normalization.

### Saccharification of delignified corncob residue

The delignified corncob residue was provided by LONGLIVE Co., Yucheng, Shandong province, China (http://www.longlive.cn/). Alkaline extraction of the lignin from corncob residues was performed and left the delignified corncob residue. The crude enzymes were collected at 144 h, and we removed the mycelia and residual medium. The saccharification reaction was implemented at 50 °C in a 5 ml Eppendorf tube containing a mixture of 0.1 g DCCR (5 %), 1 % NaN_3_ and 10 FPU/g glucan per crude enzyme with added pH 4.8 sodium acetate buffer to increase total volume to 2 ml. The glucose release was measured using the SBA-40C biological sensor (Shandong, China). The experiments were performed in three biological repetitions.

### Statistics

We performed one-tail *t*-Student tests with equal variance using the software Microsoft Office 2010 Excel. All mean values, standard deviations as well as *p* values were calculated in the quantitative analyses throughout this study.

## Additional files

10.1186/s13068-016-0491-4 Domain structure of BGL1, BGL4, BGL5.
10.1186/s13068-016-0491-4 Cellobiose hydrolysis activity assays. The culture supernatants of the BGL(X) over-expression mutants and the parental strain RE-10 were sampled at 120 and 144 h. β-glucosidase activity was measured with the cellobiose as the substrate. Data are the means of three biological replicates and error bars show the standard deviation.
10.1186/s13068-016-0491-4 HPLC assays for the released sugars. The released sugars during the fermentation for all strains were collected, and analyzed by HPLC to quantify the amount of glucose (red) and cellobiose (blue).
10.1186/s13068-016-0491-4 qPCR analysis of the transcription change of *cbh1*and *eg1* The transcript abundance of *cbh1* (A) and *eg1* (B) under cellulose induction 4 h, 24 h, 48 h and 72 h in mutants and RE-10 were analyzed.
10.1186/s13068-016-0491-4 qPCR analysis of the expression pattern of *bgl1*, *bgl4*, and *bgl5* The transcript abundance of *bgl1*, *bgl4*, and *bgl5* under glucose (4 h), no carbon source (2 h), cellulose (4 h) conditions in *P*. *oxalicum* wild type strain 114-2 were analyzed.
10.1186/s13068-016-0491-4 Primers used in this study.

## Abbreviations

WT: Wild type
BGL/*bgl*: β-Glucosidase
DCCR: delignified corncob residue
Cdt: Cellodextrin transporter
REXRN: reconstruction of expression regulatory network
GH family: glucoside hydrolase family
*p*NPG: 4-Nitrophenyl-β-d-glucopyranoside
LPMO: lytic polysaccharide monooxygenase
SDS-PAGE: sodium dodecyl sulfate polyacrylamide gel electrophoresis
HPLC: high-performance liquid chromatography
