# Supplementary figures and images for: Production of a high-efficiency cellulase complex via β-glucosidase engineering in Penicillium oxalicum
Source: Biotechnol Biofuels. 2016 Mar 31;9:78. doi: 10.1186/s13068-016-0491-4 (PMC4815182; doi:10.1186/s13068-016-0491-4)

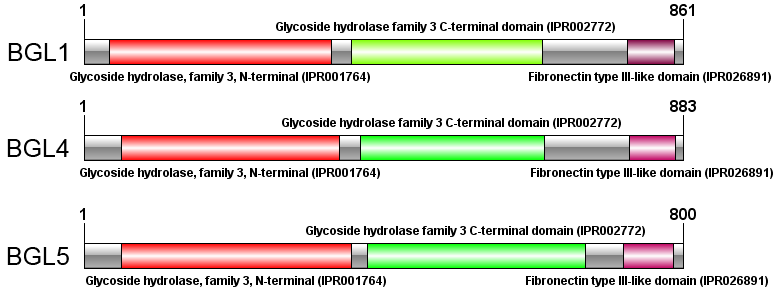

Supplement: Supplementary file 1 — 10.1186/s13068-016-0491-4 Domain structure of BGL1, BGL4, BGL5. [file 13068_2016_491_MOESM1_ESM.tif]

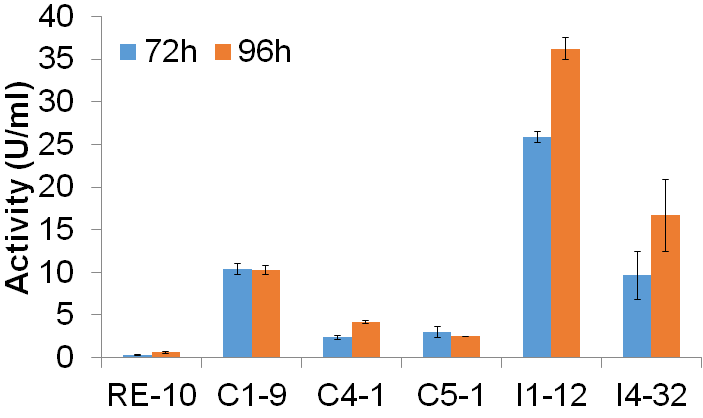

Supplement: Supplementary file 2 — 10.1186/s13068-016-0491-4 Cellobiose hydrolysis activity assays. The culture supernatants of the BGL(X) over-expression mutants and the parental strain RE-10 were sampled at 120 and 144 h. β-glucosidase activity was measured with the cellobiose as the substrate. Data are the means of three biological replicates and error bars show the standard deviation. [file 13068_2016_491_MOESM2_ESM.tif]

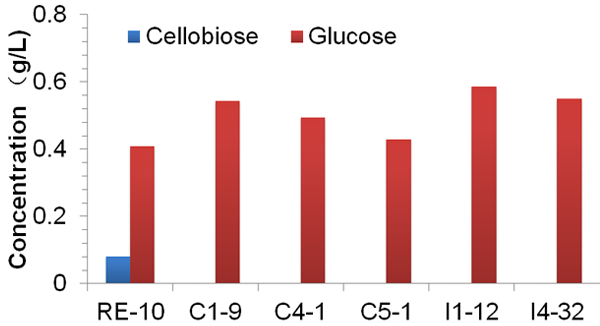

Supplement: Supplementary file 3 — 10.1186/s13068-016-0491-4 HPLC assays for the released sugars. The released sugars during the fermentation for all strains were collected, and analyzed by HPLC to quantify the amount of glucose (red) and cellobiose (blue). [file 13068_2016_491_MOESM3_ESM.tif]

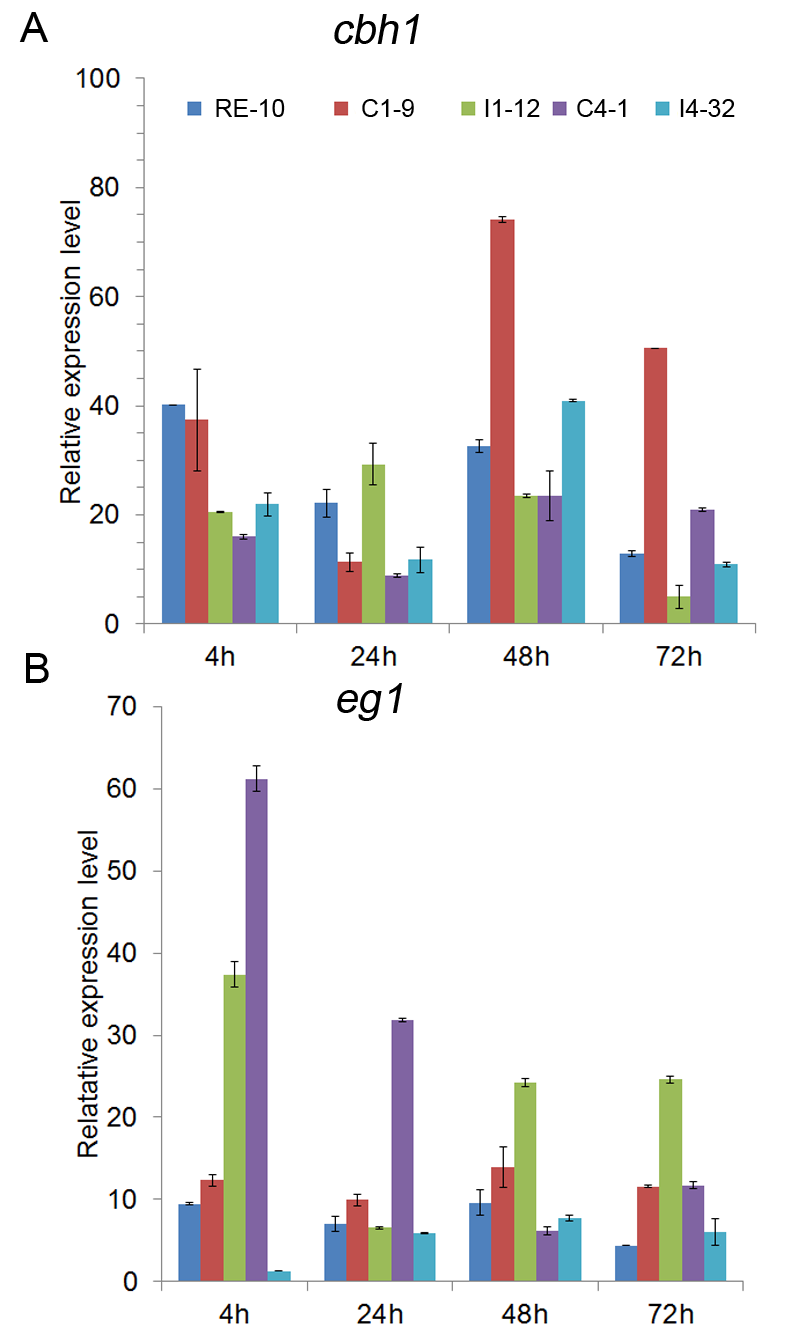

Supplement: Supplementary file 4 — 10.1186/s13068-016-0491-4 qPCR analysis of the transcription change of cbh1and eg1 The transcript abundance of cbh1 (A) and eg1 (B) under cellulose induction 4 h, 24 h, 48 h and 72 h in mutants and RE-10 were analyzed. [file 13068_2016_491_MOESM4_ESM.tif]

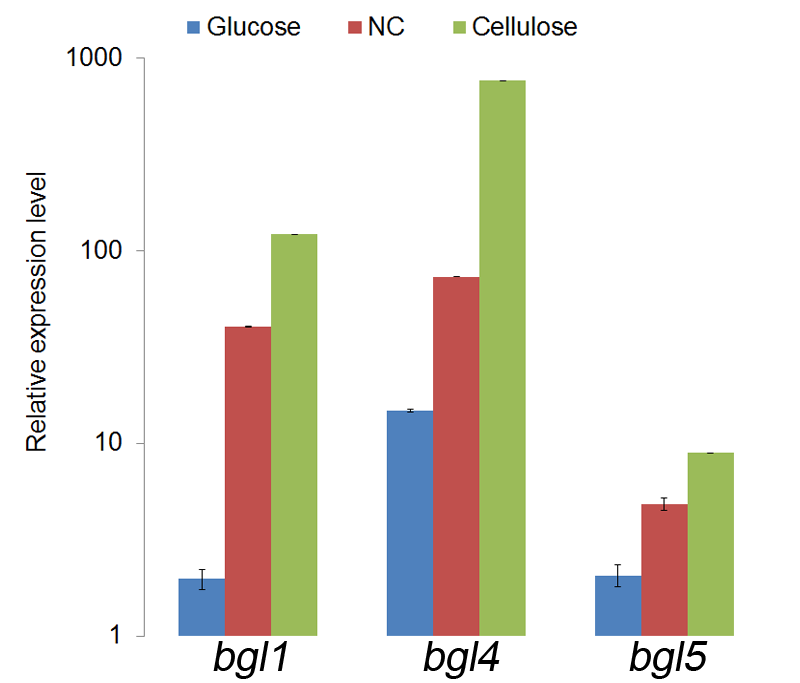

Supplement: Supplementary file 5 — 10.1186/s13068-016-0491-4 qPCR analysis of the expression pattern of bgl1, bgl4, and bgl5 The transcript abundance of bgl1, bgl4, and bgl5 under glucose (4 h), no carbon source (2 h), cellulose (4 h) conditions in P. oxalicum wild type strain 114-2 were analyzed. [file 13068_2016_491_MOESM5_ESM.tif]
