# Supplementary material for: Production of a high-efficiency cellulase complex via β-glucosidase engineering in Penicillium oxalicum
Source: Biotechnol Biofuels. 2016 Mar 31;9:78. doi: 10.1186/s13068-016-0491-4 (PMC4815182; doi:10.1186/s13068-016-0491-4)
Supplement: Supplementary file 6 — 10.1186/s13068-016-0491-4 Primers used in this study. [file 13068_2016_491_MOESM6_ESM.docx]

Additional file 2 Table 1**.** Primers used in this study.

| Primer name | Sequence (5′-3′) |
| --- | --- |
| Primers for *bgl*(*x*) overexpression in WT | |
| DF | CGCCGATTGCCTTGATACGT |
| DF2 | CTGGGTTTCTTGCCTGAGTT |
| DR | GTTTGCGATATGATGGAGGG |
| HPHsF | cgacgttaactgatattgaa |
| HPHsR | CGCAAACTTCTCGCCTTATC |
| Bgl1F | GACCCCTCCATCATATCGCAAACATGAAGCTCGAGTGGCTGGAAGC |
| Bgl1R | TCCTTCAATATCAGTTAACGTCGTCTTTCCACGAAGCATACGACAT |
| Bgl2F | CCCTCCATCATATCGCAAACATGGGATCGATAGAACAATC |
| Bgl2R | TTCAATATCAGTTAACGTCGGAAACGGAATCCTTACCTAC |
| Bgl3F | CCCTCCATCATATCGCAAACATGCGGATCCCTATTGCCTT |
| Bgl3R | TTCAATATCAGTTAACGTCGGATAATCCCAGACAGAAAGG |
| Bgl4F | GACCCCTCCATCATATCGCAAACATGAGGAGCTCAACGACGGTTCT |
| Bgl4R | TCCTTCAATATCAGTTAACGTCGTCCACAGGCTACGAAGTGATTTG |
| Bgl5F | CCCTCCATCATATCGCAAACATGATTGTCACCAAGGAGTT |
| Bgl5R | TTCAATATCAGTTAACGTCGCTCTGCTGTCTCCGATACTT |
| Bgl6F | CCCTCCATCATATCGCAAACATGGCGGTTTGGCTGCAGCT |
| Bgl6R | TTCAATATCAGTTAACGTCGTTCTCGGCGTTTATTCTCAA |
| Bgl7F | CCCTCCATCATATCGCAAACATGTCAAACTCATTCGATGT |
| Bgl7R | TTCAATATCAGTTAACGTCGACTGATTCTCATTTCTGGGT |
| Bgl8F | CCCTCCATCATATCGCAAACATGGACTTGAAAGCCGTGGA |
| Bgl8R | TTCAATATCAGTTAACGTCGAGCAGTGCCAACCTTTCCTT |
| Bgl9F | CCCTCCATCATATCGCAAACATGTCCTTCAATTCTGGATC |
| Bgl9R | TTCAATATCAGTTAACGTCGCCTGTGACCCATCGGAGTTG |
| Primers for over-expression *bgl*(*x*) in RE-10 | |
| SurF | GTCGACGTGCCAACGCCA |
| SurR | GTCGACGTGAGAGCATGCAATTCC |
| Bgl1-SurR | TGGCGTTGGCACGTCGACTTCCACGAAGCATACGACAT |
| Bgl4-SurR | TGGCGTTGGCACGTCGACACAGGCTACGAAGTGATTTG |
| Bgl5-SurR | TGGCGTTGGCACGTCGACCTCTGCTGTCTCCGATACTT |
| Pbgl2F | TGACACACTGGGGTCGCATTG |
| Pbgl2R | CTTGGCGAAGTCGATTGGAAC |
| IBg11-F | GTTCCAATCGACTTCGCCAAGATGAAGCTCGAGTGGCTGG |
| IBg14-F | GTTCCAATCGACTTCGCCAAGATGAGGAGCTCAACGACGG |
| Bgl2nestF | ACCACTCTGCCACCAACACC |
| Primers for q-PCR | |
| Bgl1F | CACCAACACCGGCTCAGTTA |
| Bgl1R | GGACATCCCAGTTGGACAGAT |
| Bgl2F | CTCGGTGCTCGGATACAACA |
| Bgl2R | GGCTGATGCGTACACGTTTGA |
| Bgl3F | ATGAGGATGGCGTTCGTGTC |
| Bgl3R | ACCACCTGAATGCCGAACTG |
| Bgl4F | TGACCGAATCCACCTCCTGC |
| Bgl4R | AACCTCCTTGGCGGACCTGA |
| Bgl5F | GCCGAAGTTGCCCAGCTCTA |
| Bgl5R | CAACCACATCCCATGTACTCAA |
| Bgl6F | GTATCTGGGACGTTGTCACGC |
| Bgl6R | GAGACCAGAGTATCGGCAACG |
| Bgl7F | GGTGTCCTTCGGCTGAGTATGT |
| Bgl7R | GCCACTCCAATAGCTCGCTTAA |
| Bgl8F | CTTTGCGTGGACATTTACCGA |
| Bgl8R | GCCGACTGCTTGGGATAACG |
| Bgl9F | CCCGAACACGTTGCAGAACA |
| Bgl9R | TCCGGGATAGGGTCATCTGT |
